# Alteration of Visuospatial System as an Early Marker of Cognitive Decline: A Double-Center Neuroimaging Study

**DOI:** 10.3389/fnagi.2022.854368

**Published:** 2022-06-10

**Authors:** Dalida Borbala Berente, Janos Zsuffa, Tom Werber, Mate Kiss, Anita Drotos, Anita Kamondi, Gabor Csukly, Andras Attila Horvath

**Affiliations:** ^1^School of PhD Studies, Semmelweis University, Budapest, Hungary; ^2^Neurocognitive Research Center, National Institute of Mental Health, Neurology and Neurosurgery, Budapest, Hungary; ^3^Department of Family Medicine, Semmelweis University, Budapest, Hungary; ^4^Faculty of Medicine, Semmelweis University, Budapest, Hungary; ^5^Siemens Healthcare, Budapest, Hungary; ^6^Department of Neurology, Semmelweis University, Budapest, Hungary; ^7^Research Group of Clinical Neuroscience and Neuroimaging, Department of Psychiatry and Psychotherapy, Semmelweis University, Budapest, Hungary; ^8^Department of Anatomy, Histology and Embryology, Semmelweis University, Budapest, Hungary

**Keywords:** amnestic mild cognitive impairment (aMCI), neuropsychology, visuospatial network, structural neuroimaging, functional MRI (fMRI), functional connectivity

## Abstract

Amnestic-type mild cognitive impairment (a-MCI) represents the prodromal phase of Alzheimer's disease associated with a high conversion rate to dementia and serves as a potential golden period for interventions. In our study, we analyzed the role of visuospatial (VS) functions and networks in the recognition of a-MCI. We examined 78 participants (32 patients and 46 controls) in a double-center arrangement using neuropsychology, structural, and resting-state functional MRI. We found that imaging of the lateral temporal areas showed strong discriminating power since in patients only the temporal pole (*F* = 5.26, *p* = 0.034) and superior temporal gyrus (*F* = 8.04, *p* < 0.001) showed reduced cortical thickness. We demonstrated significant differences between controls and patients in various neuropsychological results; however, analysis of cognitive subdomains revealed that the largest difference was presented in VS skills (*F* = 8.32, *p* < 0.001). Functional connectivity analysis of VS network showed that patients had weaker connectivity between the left and right frontotemporal areas, while stronger local connectivity was presented between the left frontotemporal structures (FWE corrected *p* < 0.05). Our results highlight the remarkable potential of examining the VS system in the early detection of cognitive decline. Since resting-state setting of functional MRI simplifies the possible automatization of data analysis, detection of VS system alterations might provide a non-invasive biomarker of a-MCI.

## Introduction

As the age of the world's population increases, the number of people living with major neurocognitive disorders is growing. Worldwide, nearly 50 million people are affected by dementia and this number is expected to triple by 2050 (Brookmeyer et al., 2007; Doody et al., 2010). Alzheimer's disease (AD) is the primary cause of the cognitive decline. The main symptoms of AD are the loss of episodic memory and orientation, the impairment of social functioning, behavioral changes, and communication difficulties. Since AD is the 6th most common cause of death—due to its large prevalence and significant medical burden—it has become one of the greatest scientific and social challenges of the 21st century (Livingston et al., 2017).

Despite the tremendous research effort, we are still not able to decrease the progression of cognitive decline caused by neurodegenerative disorders. The major reason is that by the time the clinical symptoms become unequivocal, the pathophysiological process that started many years or decades before the diagnosis of AD already causes irreversible damage to the brain (Wilson et al., 2011). Thus, the aging research is advancing rapidly toward the stage of the earliest identification of prodromal phases which might provide a critical opportunity for therapeutic interventions (Sperling et al., 2011). Dementia is preceded by mild cognitive impairment (MCI), which is an early stage of memory loss in individuals who are still capable to live independently (Petersen et al., 2009). Follow-up studies demonstrated that more than 50% of MCI subjects converted to dementia in 4 years. The amnestic subtype of MCI (a-MCI) has the highest risk of progression to AD, constituting a prodromal stage of AD (Petersen et al., 2009). The most common early warning signs of MCI are short-term memory loss or difficulty in finding words. Some reports also suggest remarkable impairment of visuospatial (VS) abilities and their diagnostic value in cognitive assessment in the earliest stages of MCI (Iachini et al., 2009; Mitolo et al., 2013; Choi et al., 2020; Wasserman et al., 2020).

Unfortunately, current data report that MCI and mild dementia are frequently (~50–60%) undiagnosed (Amjad et al., 2018). A possible reason is that only 16% of adults 65 years of age or older receive regular cognitive testing (Livingston et al., 2020). Furthermore, many neuropsychological tests are not sensitive enough to recognize early cognitive deficits (Albert et al., 2011). Analysis of cerebrospinal fluid (CSF) and FDG-PET imaging can sensitively signal the presence of neurotoxic proteins even in the earliest stages. However, their use is limited, since CSF analysis is invasive and PET imaging requires the use of a radioactive pharmakon which causes logistical difficulties and expensive examination costs. Magnetic resonance imaging (MRI) is a more readily available, non-invasive alternative and it provides an ideal tool to investigate the structural and functional neural changes *in vivo*. MCI is characterized by prominent structural MRI (sMRI) and functional MRI (fMRI) alterations (Talwar et al., 2021). Structurally, the major characteristic hallmark of MCI is the atrophy of the medial temporal lobe (e.g., hippocampus, entorhinal cortex), lateral temporal cortices (e.g., superior temporal gyrus, inferior temporal gyrus, temporal pole), the posteromedial cortices (e.g., precuneus, superior parietal lobule), and frontal cortices (e.g., superior frontal gyrus, middle frontal gyrus). Functional MRI studies in MCI reported changes in the medial temporal lobe activity during encoding new visually demonstrated information. Resting-state fMRI (which is based on the low-frequency BOLD fluctuations) revealed the malfunction of the default mode network, and the posteromedial cortical region. Noticeably, reports agree that functional alterations in MCI frequently precede the appearance of structural changes (Jack et al., 2009).

Since a growing body of evidence supports the early impairment of VS functions in MCI and the usefulness of MRI in the early recognition of cognitive decline, our study aimed to analyze the functional connectivity and structural integrity of VS network in a-MCI patients with the application of sMRI and resting-state fMRI.

## Materials and Methods

### Participants

Data of 32 patients with a-MCI and 46 healthy controls (HC) (altogether 78 participants) were included in the study. The data set was formed by combining the data of cohorts collected by two independent research centers that used the same clinical and research protocols for clinical and imaging investigations: (1) the Semmelweis MCI Neuroimaging Cohort (SMNC) (data of 13 patients and 20 controls) and the (2) AlzEpi Cohort Observational Library (ACOL) (data of 19 patients and 26 controls). Data were harmonized under the egis of the Euro-Fingers Consortium (www.eufingers.com). Participants were recruited from the Department of Psychiatry and Psychotherapy, Semmelweis University (SMNC database), and from the National Institute of Mental Health, Neurology, and Neurosurgery (ACOL database). All subjects were native Hungarians.

All patients went through a detailed dementia screening protocol including medical history, psychiatric and neurological examinations, blood tests, detailed neuropsychological testing, brain sMRI, and resting-state fMRI. The diagnosis was established by a multidisciplinary medical team. MCI patients met the Petersen criteria based on subjective memory complaints reinforced by an objective cognitive test, in the absence of dementia or significant functional loss and the daily independent functions are preserved (Petersen et al., 2009). Rey Auditory Verbal Learning Test (RAVLT) was applied to identify patients with a-MCI, since it is the most frequently used test for early memory impairment and established cut-off scores are available (Petersen, 2004; Gomar et al., 2011; Csukly et al., 2016). Participants, who scored under the cut-off value both in the delayed recall subscore and in the total score of the first five trials, represented the a-MCI group (Table 1). Healthy controls had normal neurological status and neuropsychology scores, normal brain MRI, and blood results, and they had no cognitive complaints. The Hungarian Medical Research Council (reference number: 024505/2015) authorized our research. We obtained informed written consent from each participant.

**Table 1:** Applied age and education adjusted cut-off scores for the exclusion of dementia and for the detection of amnestic-type MCI (a-MCI).

**MMSE cut-off scores for the exclusion of dementia**								
Education\age	50–54	55–59	60–64	65–69	70–74	75–79	80–84	85+
5–8 years	23	23	23	23	23	21	21	17
9–12 years	25	25	25	25	24	24	21	21
>12 years	27	27	27	27	25	25	25	24
**^a^RAVLT Sum 5 cut-off scores for a-MCI**								
**Age**	**Score**							
50–59	39							
60–69	35							
70+	29							
**^b^RAVLT 7 cut-off scores for a-MCI**								
**Age**	**Score**							
50–59	6							
60–69	5							
70+	4							

*a-MCI, amnestic mild cognitive impairment; MMSE, mini mental state examination; RAVLT, rey auditory verbal learning task.*

a *The summarized number of learned words in the first five trials (maximum number is 75).*

b *The number of recalled words after 30 min delay (maximum number is 15)*.

Subjects with dementia were excluded from the study based on their age and education and standardized Mini-Mental Examination Scores (MMSE) (Table 1). Further exclusion criteria were also applied including conditions compromising cognitive functions except for a-MCI as the following: prior central nervous infection, clinically significant brain lesions (cortical stroke, severe periventricular white matter disease, and white matter infarcts), head trauma with loss of consciousness, demyelinating conditions, hydrocephalus, untreated vitamin B12 deficiency, hypothyroidism, syphilis, HIV infection, major depression, schizophrenia, electroconvulsive therapy, renal insufficiency, liver disease, significant systemic medical illness, alcohol or substance dependency, psychoactive drugs affecting cognitive functions.

### Neuropsychological Examination

Neuropsychological tests were administered by trained neuroscientists, neurologists, or neuropsychologists. MMSE test (max 30 points) (Creavin et al., 2016) was applied for the exclusion of patients with dementia since it is the most frequently used standard test in dementia research. While many studies applied 26 as the cut-off score for clinically evident dementia, we performed a widely accepted method with the use of the educational background and age of subjects (Table 1) (Strauss, 2006).

The validated Hungarian version of the Addenbrooke Cognitive Examination (Kaszás and Fekete, 2020) was applied to assess global cognitive performance (max 100 points) and the major subdomains of cognition including orientation (10 points), attention (8 points), memory (35 points), verbal fluency (14 points), language (28 points), and VS abilities (5 points). While a-MCI patients have normal MMSE scores, studies indicate that ACE scores might already sensitively signal impaired cognitive performance (Crawford et al., 2012).

The validated Hungarian version of RAVLT (Kónya et al., 1995) was administered to objectively assess memory complaints according to the Petersen criteria. Previous studies revealed its prominent sensitivity in the detection of a-MCI (Alladi et al., 2006) due to the early involvement of verbal-learning oriented memory functions. Subjects need to memorize a list of 15 words (list A) and recall it with 5 repetitions (RAVLT sum-5: immediate recalls described with the total number of correct words) and then another 15 words are presented once (list B) followed by a recall. Later, list A should be recalled without repetition and the same task is required 30 min later (RAVLT 7: delayed recall described with the total number of correct words).

Trail-Making test A (TMT-A) was used to measure executive functions and attention, while B (TMT-B) was applied to estimate cognitive flexibility (Bowie and Harvey, 2006). Participants are asked to connect numbers in ascending order (test A) and numbers and letters in alphabetic order (1-A, 2-B, etc.). Results are described in the required time (in seconds).

### Structural and Functional MRI Data Acquisition

All subjects were examined using brain Magnetic Resonance Imaging (MRI), producing high-resolution structural images, which are used for further processing analysis. At the two sites of our study, two different scanners were used. At the National Institute of Mental Health, Neurology, and Neurosurgery, a Siemens Magnetom Verio 3T scanner (Siemens Healthcare, Erlangen, Germany) was used with the standard 12 channels head receiver head coil. The protocol consisted of T1-weighted 3D MPRAGE (magnetization prepared rapid gradient echo) anatomical imaging (TR = 2,300 ms; TE = 3.4 ms; TI = 100 ms; flip angle: 12°; voxel size: 1.0 × 1.0 × 1.0 mm). The second measurement was a resting-state functional MRI, an EPI-based MRI sequence (TR = 2,000 ms; TE = 30 ms; flip angle = 79°; voxel size = 3 × 3 × 3 mm). The fMRI scan was 10 min long, while patients were laying on the table with closed eyes.

On the second site, image acquisitions were done at the MR Research Center, Semmelweis University on a 3 Tesla Philips Achieva whole-body MRI scanner (Philips Medical Systems, Best, The Netherlands) equipped with an 8-channel SENSE head coil. The high-resolution, whole-brain anatomical images were obtained using a T1 weighted three-dimensional spoiled gradient echo (T1W 3D Turbo Field Echo) sequence. About 180 contiguous slices were acquired from each subject with the following imaging parameters: TR (time resolution) = 9.7 ms; TE (echo time) = 4.6 ms; flip angle = 8°; FOV (field-of-view) of 240 mm × 240 mm; voxel size of 1.0 × 1.0 × 1.0 mm. The “resting-state” part of the fMRI acquisition took ~8.5 min. During that time, subjects were instructed to fixate on a cross in the center of the screen. Subjects were briefed on whether they fell asleep during the recording process, and no subject reported doing so. Head motion was minimized using foam padding. Functional images were acquired using a T2^*^ weighted echo-planar imaging (EPI) sequence with the following parameters: TR = 2.0 s; TE = 30 ms; flip angle = 70°, FOV of 240 mm × 240 mm; voxel size of 3.0 × 3.0 × 4.0 mm; number of slices = 36.

Both protocols consisted of a T2-, diffusion-, and a FLAIR-weighted sequence to identify the possible pathological lesions.

### Imaging Analysis

T1-weighted 3D anatomical images were used for cortical reconstruction and volumetric segmentation performed by Freesurfer 6.0 image analysis software which is documented and freely available for download online (http://surfer.nmr.mgh.harvard.edu/). The technical details of the reconstruction procedures are demonstrated in previous reports (Csukly et al., 2016). Without changing the pipeline, we used the “recon-all” processing stream with the default parameters to create a cortical surface model. Particularly, image processing involves motion correction, removal of non-brain tissue, using a hybrid watershed/surface deformation process, automated Talairach transformation, segmentation of the subcortical white matter and deep gray matter volumetric structures, intensity normalization, tessellation of the gray matter, white matter boundary, automated topology correction, and surface deformation following intensity gradients to optimally place gray/white and gray/cerebrospinal fluid borders at the location where the greatest shift in intensity defines the transition to the other tissue class. When the cortical models were finished, Freesurfer performed numerous deformable processes for following data processing and analysis. Steps included surface inflation, registration to a spherical atlas being utilized individual cortical folding patterns to match cortical geometry across subjects, parcellation of the cerebral cortex into units based on gyral and sulcal structure, and creation of a variety of surface-based data including maps of curvature and sulcal depth. Finally, cortical models and the results of segmentation were quality checked and manually corrected on every subject. Cortical thickness (described in mm) and cortical volume were extracted from the analysis for numerous cortical areas.

CONN MATLAB toolbox was used for resting-state fMRI data analysis (Whitfield-Gabrieli and Nieto-Castanon, 2012). We used the standard preprocessing pipeline on both data set, which includes the functional realignment and unwarp, slice-time correction (interleaved at Siemens' scanner data ascending at Philipps' scanner data), outlier detection (ART-based identification of outlier scans for scrubbing), direct functional and structural segmentation and normalization (simultaneous Gray/White/CSF segmentation and MNI normalization), and spatial smoothing. After the preprocessing, we ran an additional quality check to qualify that segmentation was appropriate. We applied a band-pass filter between 0.008 and 0.09 Hz to eliminate the physiological-based artifacts and the unrelated part of the measured signal. Finally, we used linear regression to filter out/eliminate white matter, CSF signal, and the effect of realignment and scrubbing.

**Figure 1:**
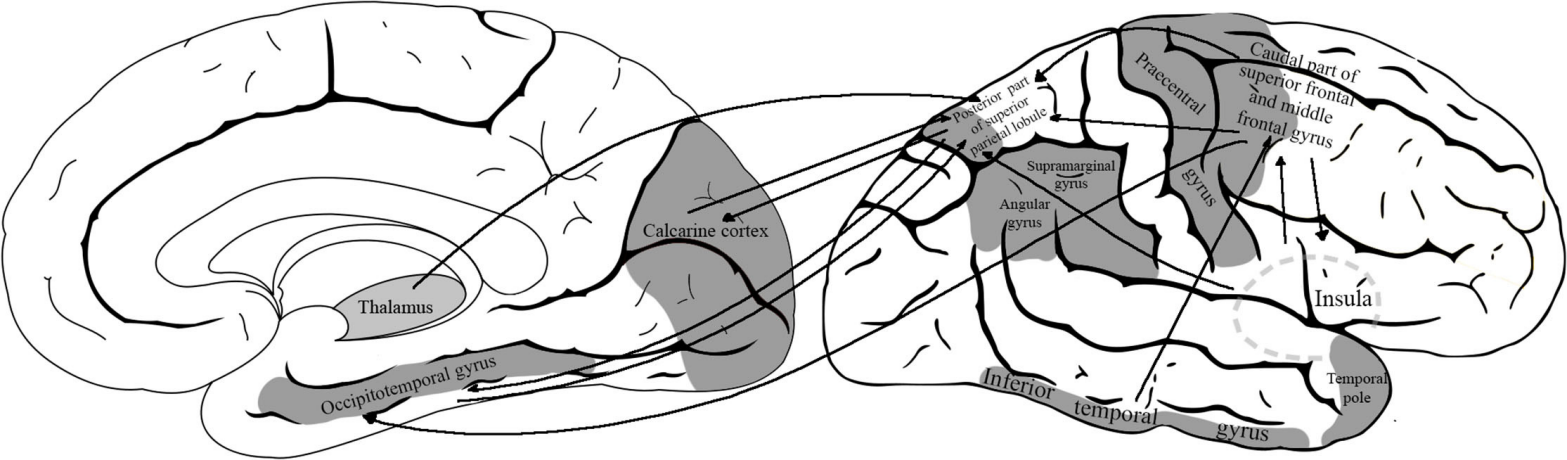
The model of visuospatial network with the defined region-of-interest cortical areas. The analyzed network included the following areas: calcarine cortex, posterior part of superior parietal lobule, inferior temporal gyrus, occipitotemporal gyri, supramarginal gyrus, angular gyrus, caudal part of middle frontal and superior frontal gyri, precentral gyrus, temporal pole, and insula.

The definition region of interest areas of VS network was based on the consensus of existing literature. The process of visuospatial information gathering circles around three key components: spatial perception, recognition, and analysis of visual input (Graaf et al., 2010). Many studies (Postle et al., 2000; Formisano et al., 2002; Klingberg et al., 2002; Culham et al., 2006; Scherf et al., 2006; Trojano and Conson, 2008) focusing on visuospatial tasks utilize fMRI studies to converge on a conclusion that visuospatial network activation consistently involves activation of some parietal, temporal, and frontal regions. There is an agreement in the literature regarding the role of the following regions (Ghaem et al., 1997; Postle et al., 2000; Formisano et al., 2002; Klingberg et al., 2002; Culham et al., 2006; Ricciardi et al., 2006; Scherf et al., 2006; Suchan et al., 2006; Trojano and Conson, 2008; Zimmer, 2008; Thakral and Slotnick, 2009; Graaf et al., 2010): calcarine cortex, posterior part of superior parietal lobule, inferior temporal gyrus, occipitotemporal gyri, supramarginal gyrus, angular gyrus, caudal part of middle frontal and superior frontal gyri, precentral gyrus, temporal pole, and insula (Figure 1). Data also show that the subdominant hemisphere (usually right) is involved more in the organization of VS functions (Ng et al., 2000; Corballis, 2003).

To reveal the functional connectivity at every subject, and group level as well, we used Seed-base connectivity (SBC) analysis (Sheline and Raichle, 2013) which characterizes the connectivity patterns with a pre-defined (above-mentioned) ROI-s. SBC maps are computed as the Fischer-transformed bivariate correlation coefficients between an ROI BOLD time series and each voxel BOLD time series. Age and sex were added as covariates to the analysis. A paired *t*-test (the contrast was: controls > aMCI) was applied to depict the functional differences between the study groups. Parametric statistics used are as follows (Worsley et al., 1996): cluster threshold: *p* < 0.05, cluster-size: p-FDR corrected, voxel threshold: *p* < 0.001 uncorrected.

### Statistics on Epidemiology, Neuropsychology, and Structural Neuroimaging

IBM SPSS 20 software (https://www.ibm.com/support/pages/ibm-spss-statistics-20-documentation) was applied for all statistical analyses other than functional MRI. Epidemiological factors (sex, age, and educational level) were compared in pairwise comparisons (HC vs. a-MCI). Independent sample *t*-tests were used for continuous data with parametric distribution, while Mann–Whitney *U*-test was selected for data with non-parametric distribution (educational level). The chi-square test was applied for categorical variables (sex). Neuropsychological results and structural MRI results were compared between study groups by ANCOVA including age and sex as covariates. *P*-values were adjusted for multiple comparisons with the Benjamini–Hochberg procedure in the analysis of structural neuroimaging and neuropsychology. Effect size was reported in Cohen's d (0.2–0.5 = small, 0.5–0.8 = medium, >0.8 = large).

## Results

### Demographics and Cognitive Performance

Differences were found in the age and sex ratio among the study groups, while in the length of education of HC and a-MCI, subjects did not differ significantly (*p* = 0.142). A higher ratio of female participants was found in the HC group with the chi-square test (χ^2^ = 5.128; *p* = 0.024). The patient group represented a significantly older study population (*F* = 6.18; *p* = 0.015).

Differences were found in the total scores of all neuropsychological tests (Table 2). Many of them survived the application of Benjamini–Hochberg correction: a-MCI patients had lower MMSE score (*F* = 9.098; *p* < 0.001), lower total ACE score (*F* = 11.065; *p* < 0.001), lower RAVLT sum-5 score (*F* = 13.53; *p* < 0.001), and lower RAVLT 7 score (*F* = 11.9; *p* < 0.001). Significantly longer time for TMT-A (*F* = 4.69; *p* = 0.048) and TMT-B (*F* = 5.51; *p* = 0.021) was also demonstrated in the aMCI study group indicating weaker cognitive performance. Controls significantly outperformed a-MCI patients in VS skills (*F* = 8.32; *p* < 0.001), while study groups did not differ in other cognitive subdomains regarding the corrected *p* (*p* > 0.05). Age and sex did not have a significant modifier effect on the neuropsychological results (*p* > 0.05).

**Table 2:** Demographic and neuropsychological characteristics of study groups.

	**HC (*n*: 46)**	**a-MCI (*n*: 32)**	***p*-value**	**Effect size**
**Demographics**				
^a^Age (years)	67.63 ± 7.15	70.68 ± 9.94	0.015^*^	0.352
^b^Sex (% of females)	69.6	53.1	0.024^*^	–
^c^Education (years)	15 ± 2.53	14.43 ± 3.13	0.142	0.200
**Neuropsychology**				
^d^MMSE	28.52 ± 1.13	26.87 ± 1.62	*p* < 0.001^*^	1.181
^d^ACE Total	93.24 ± 3.29	82.31 ± 7.26	*p* < 0.001^*^	1.939
^d^ACE Orientation	9.88 ± 0.31	9.43 ± 0.8	0.029	0.742
^d^ACE Attention	7.91 ± 0.28	7.65 ± 0.86	0.134	0.407
^d^ACE Memory	30.97 ± 2.11	24.68 ± 5.47	0.03	1.517
^d^ACE Verbal fluency	11.95 ± 2.24	9.62 ± 2.87	0.021	0.905
^d^ACE Language	27.71 ± 0.54	27.125 ± 1.58	0.378	0.495
^d^ACE Visuo-spatial	4.71 ± 0.5	3.78 ± 1.12	<0.001^*^	1.072
^d^RAVLT Sum-5	48.43 ± 8.69	31.15 ± 9.4	<0.001^*^	1.909
^d^RAVLT 7	9.89 ± 2.75	4.03 ± 2.83	<0.001^*^	2.100
^d^^,^^e^^,^^f^TMT-A	39.62 ± 10.58	90.41 ± 66.98	0.008^*^	1.059
^d^^,^^e^^,^^f^TMT-B	83.13 ± 32.67	209.33 ± 0.147.31	0.003^*^	1.183

*Data are presented as means ± standard deviations. Sex is described as the % of female participants in the study groups.*

P is reported in nominal form, while * indicates significant differences where p < 0.05 following Benjamini–Hochberg correction. Effect size is given in Cohen's d (0.2–0.5 = small, 0.5–0.8 = medium, >0.8 = large).

*a-MCI, amnestic mild cognitive impairment; HC, healthy control; MMSE, Mini Mental State Examination; RAVLT, Rey Auditory Verbal Learning Task; ACE, Addenbrooke Cognitive Examination; TMT, Trail Making Test.*

a *Analyzed with independent sample t-test.*

b *Analyzed with chi-square test.*

c *Analyzed with Mann–Whitney U-test.*

d *Analyzed with ANCOVA analysis with age and sex as covariates.*

e *Missing data of 1–4 subjects.*

f *Lower scores indicate better (faster) performance*.

### Structural MR Imaging Results

**Figure 2:**
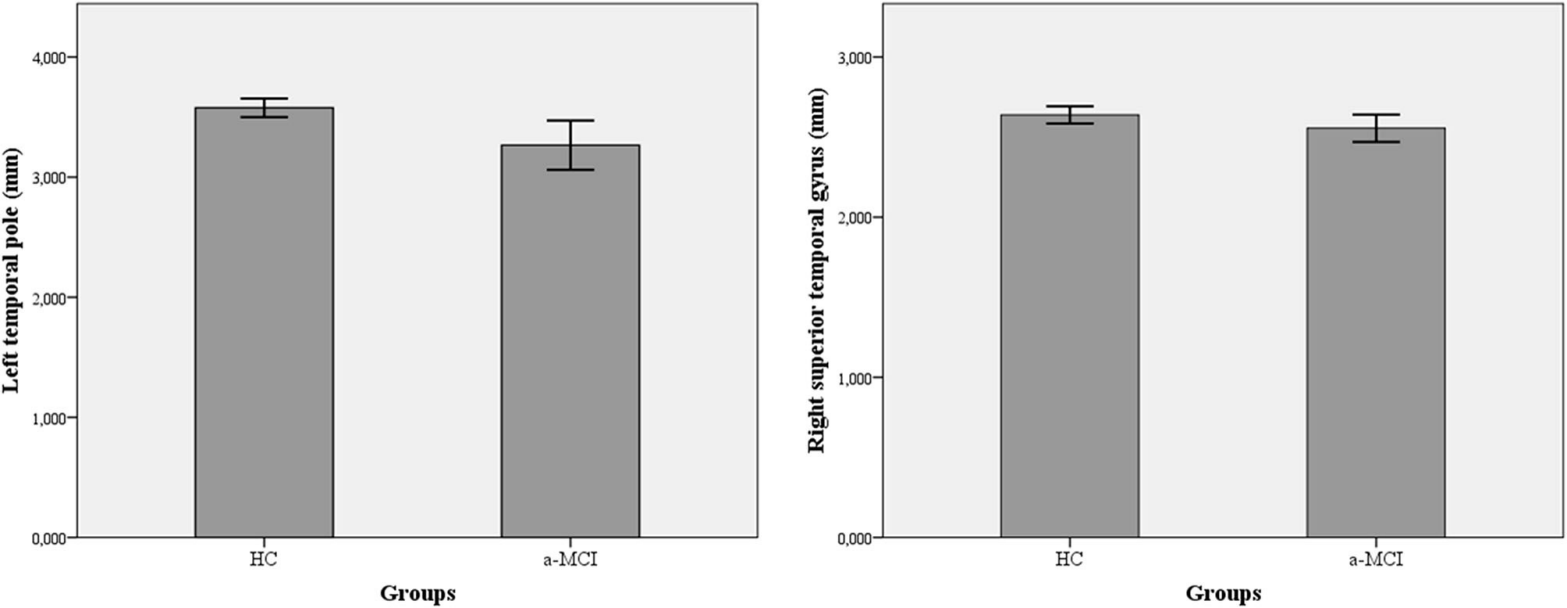
Differences in cortical thickness among amnestic-type MCI patients and healthy controls. A-MCI patients showed significant reduction in the thickness of left temporal pole and right superior temporal gyrus. a-MCI, amnestic mild cognitive impairment; HC, healthy control; LTH, left thickness; RTH, right thickness.

A significant reduction was demonstrated in the thickness of numerous cortical areas among a-MCI patients compared to HC (Table 3). The largest *F*-values were found in the thickness of the left temporal pole (*F* = 5.26) and the right superior temporal gyrus (*F* = 8.04). After the Benjamini–Hochberg correction, only the left temporal pole and right superior temporal gyrus remained significant (*p* = 0.034 and *p* < 0.001) (Figure 2). Age had a significant modifier effect on the left frontal areas (caudal middle frontal *p* = 0.049; lateral orbitofrontal *p* =0.008; medial orbitofrontal *p* =0.004; inferior frontal pars opercularis *p* =0.006; inferior frontal pars orbitalis *p* =0.024; inferior frontal pars triangularis *p* = 0.027; paracentral *p* = 0.04), right frontal areas (inferior frontal pars opercularis *p* = 0.033; inferior frontal pars orbitalis *p* = 0.005; precentral *p* = 0.016), left temporal areas (parahippocampal *p* = 0.006; superior temporal *p* = 0.003; temporal pole *p* = 0.022; entorhinal *p* = 0.009), and right temporal areas (temporal pole *p* = 0.002; superior temporal *p* = 0.0015). The largest effect of age was identified on the left side in the opercular part of the inferior frontal gyrus (*F* = 9.39) and on the right in the superior temporal gyrus (*F* = 21.81). Effect of age disappeared after Benjamini–Hochberg correction (*p* > 0.05). Sex did not have a significant modifier effect (*p* > 0.05).

**Table 3:** Cortical thickness of a-MCI and HC subjects derived from structural MRI.

**Anatomical region**	**Left hemisphere (in mm)**				**Right hemisphere (in mm)**			
	**HC**	**a-MCI**	***p***	**Effect size**	**HC**	**a-MCI**	***p***	**Effect size**
Cortex around superior temporal sulcus	2.26 ± 0.2	2.19 ± 0.2	0.51	0.396	2.36 ± 0.2	2.43 ± 0.4	0.212	0.230
Caudal anterior cingulate gyrus	2.61 ± 0.3	2.63 ± 0.3	0.64	0.074	2.5 ± 0.2	2.42 ± 0.3	0.004	0.353
Caudal middle frontal gyrus	2.43 ± 0.1	2.28 ± 0.3	0.003	0.664	2.42 ± 0.2	2.38 ± 0.2	0.017	0.241
Cuneus	1.75 ± 0.1	1.81 ± 0.2	0.566	0.345	1.81 ± 0.1	1.8 ± 0.1	0.190	0.077
Entorhinal cortex	3.35 ± 0.3	3.15 ± 0.5	0.003	0.515	3.52 ± 0.4	3.36 ± 0.4	0.002	0.385
Fusiform gyrus	2.66 ± 0.1	2.52 ± 0.2	0.004	0.830	2.7 ± 0.1	2.59 ± 0.2	0.006	0.667
Inferior parietal lobule	2.3 ± 0.11	2.22 ± 0.2	0.005	0.559	2.31 ± 0.1	2.3 ± 0.2	0.180	0.062
Inferior temporal gyrus	2.65 ± 0.1	2.56 ± 0.2	0.005	0.588	2.73 ± 0.1	2.64 ± 0.2	0.005	0.566
Isthmus of cingulate gyrus	2.21 ± 0.2	2.15 ± 0.2	0.052	0.333	2.23 ± 0.2	2.17 ± 0.2	0.003	0.291
Lateral occipital cortex	2.11 ± 0.1	2.07 ± 0.2	0.046	0.274	2.14 ± 0.1	2.07 ± 0.2	0.115	0.424
Lateral orbitofrontal cortex	2.54 ± 0.1	2.48 ± 0.2	0.002	0.339	2.57 ± 0.2	2.49 ± 0.2	0.012	0.483
Lingual gyrus	1.92 ± 0.1	1.85 ± 0.1	0.095	0.595	1.97 ± 0.1	1.88 ± 0.2	0.009	0.663
Medial orbitofrontal cortex	2.33 ± 0.1	2.25 ± 0.2	0.005	0.608	2.34 ± 0.2	2.31 ± 0.2	0.004	0.187
Middle temporal gyrus	2.66 ± 0.1	2.55 ± 0.2	0.08	0.609	2.73 ± 0.1	2.72 ± 0.2	0.014	0.067
Para-hippocampal gyrus	2.7 ± 0.3	2.61 ± 0.2	0.011	0.379	2.63 ± 0.3	2.57 ± 0.3	0.005	0.222
Paracentral lobule	2.27 ± 0.1	2.2 ± 0.2	0.019	0.430	2.35 ± 0.2	2.25 ± 0.2	0.05	0.500
Inferior frontal gyrus pars opercularis	2.41 ± 0.1	2.31 ± 0.2	0.004	0.527	2.41 ± 0.1	2.41 ± 0.1	0.165	0
Inferior frontal gyrus pars orbitalis	2.53 ± 0.2	2.48 ± 0.4	0.006	0.170	2.64 ± 0.2	2.6 ± 0.2	0.023	0.202
Inferior frontal gyrus pars triangularis	2.26 ± 0.1	2.18 ± 0.3	0.197	0.395	2.31 ± 0.1	2.32 ± 0.2	0.171	0.072
Pericalcarine cortex	1.52 ± 0.1	1.58 ± 0.2	0.118	0.404	1.55 ± 0.1	1.59 ± 0.2	0.537	0.229
Postcentral gyrus	1.91 ± 0.1	1.89 ± 0.1	0.066	0.166	1.91 ± 0.1	1.89 ± 0.2	0.005	0.123
Posterior cingulate gyrus	2.4 ± 0.1	2.15 ± 0.6	0.014	0.626	2.39 ± 0.2	2.31 ± 0.2	0.36	0.453
Precentral gyrus	2.43 ± 0.2	2.35 ± 0.2	0.173	0.442	2.41 ± 0.1	2.33 ± 0.2	0.002	0.420
Precuneus	2.2 ± 0.1	2.15 ± 0.2	0.035	0.327	2.24 ± 0.1	2.21 ± 0.1	0.006	0.230
Rostral anterior cingulate gyrus	2.74 ± 0.2	2.61 ± 0.2	0.071	0.616	2.83 ± 0.2	2.73 ± 0.2	0.355	0.465
Rostral middle frontal gyrus	2.25 ± 0.1	2.22 ± 0.2	0.242	0.219	2.24 ± 0.1	2.27 ± 0.2	0.031	0.235
Superior frontal gyrus	2.6 ± 0.1	2.49 ± 0.2	0.002	0.656	2.58 ± 0.1	2.51 ± 0.2	0.004	0.463
Superior parietal lobule	2.06 ± 0.1	2.01 ± 0.2	0.018	0.18	2.02 ± 0.1	2 ± 0.2	0.011	0.135
Superior temporal gyrus	2.57 ± 0.2	2.46 ± 0.3	0.003	0.429	2.62 ± 0.2	2.42 ± 0.2	<0.001^*^	1
Supra-marginal gyrus	2.34 ± 0.1	2.27 ± 0.4	0.079	0.235	2.37 ± 0.1	2.38 ± 0.3	0.217	0.048
Frontal pole	2.58 ± 0.2	2.59 ± 0.3	0.886	0.041	2.56 ± 0.3	2.6 ± 0.2	0.702	0.159
Temporal pole	3.48 ± 0.2	3.32 ± 0.5	0.001^*^	0.414	3.6 ± 0.4	3.55 ± 0.3	0.004	0.145
Transverse temporal cortex	2.08 ± 0.2	2.01 ± 0.3	0.016	0.286	2.13 ± 0.2	2.11 ± 0.3	0.013	0.068
Insular cortex	2.88 ± 0.2	2.74 ± 0.4	0.222	0.471	2.89 ± 0.2	2.81 ± 0.3	0.130	0.367

*a-MCI, amnestic mild cognitive impairment; HC, healthy control.*

*Data are presented as means ± standard deviations.*

P is reported in nominal form, while * indicates significant differences, where p < 0.05 following Benjamini–Hochberg correction.

*Effect size is given in Cohen's d (0.2–0.5 = small, 0.5–0.8 = medium, >0.8 = large).*

### Functional Connectivity Differences

**Figure 3:**
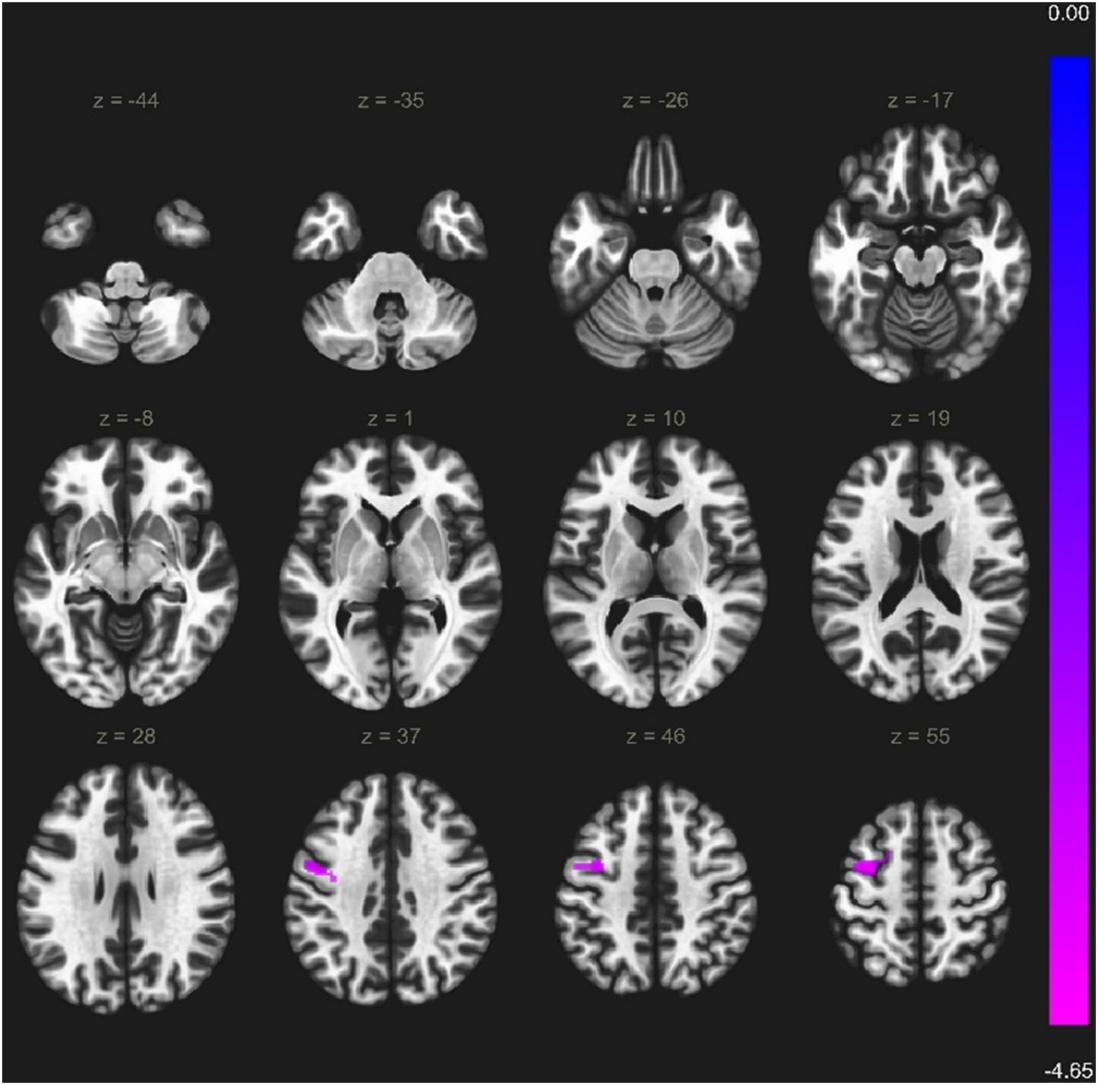
Significantly weaker connections (cyan color) between right middle frontal gyrus (seed) and the left precentral gyrus, left middle frontal gyrus and left superior frontal gyrus. Voxel level threshold *p* < 0.001 (uncorrected), cluster level threshold *p* < 0.05 (FEW corrected). Contrast: aMCI > Healthy controls. Color bar depicts the Fischer-transformed correlation values.

**Figure 4:**
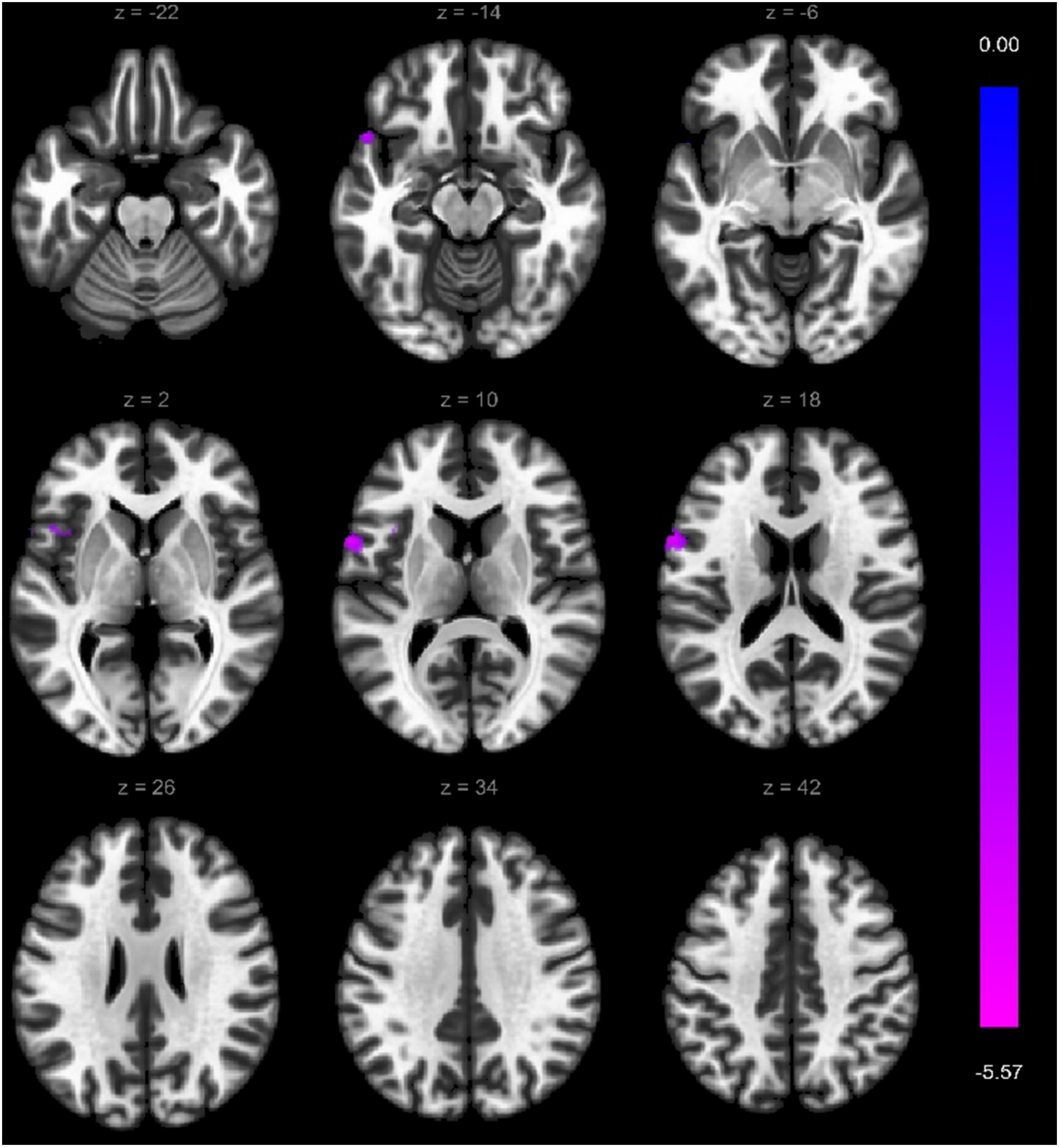
Significantly weaker connections (cyan color) between right superior frontal gyrus (seed) and the left precentral gyrus, left inferior temporal gyrus, left frontal operculum cortex, and left temporal pole. Voxel-level threshold *p* < 0.001 (uncorrected) and cluster-level threshold *p* < 0.05 (FEW corrected). Contrast: aMCI > Healthy controls. Color bar depicts the Fischer-transformed correlation values.

**Figure 5:**
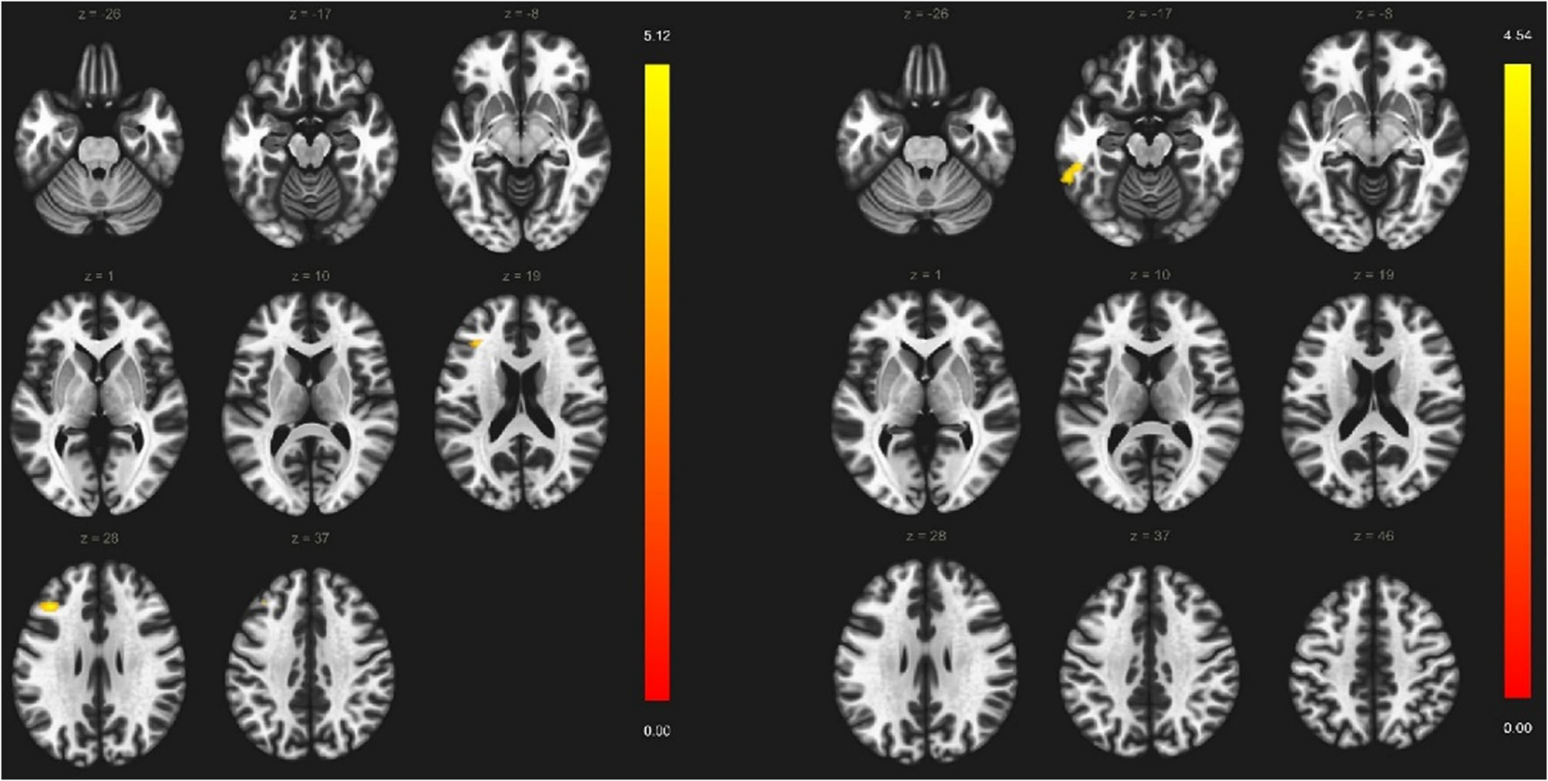
Significantly stronger connections (yellow color) among left inferior temporal gyrus (seed), the left middle frontal gyrus, and left inferior frontal gyrus. Voxel-level threshold *p* < 0.001 (uncorrected) and cluster-level threshold *p* < 0.05 (FEW corrected). Contrast: aMCI > Healthy controls. Color bar depicts the Fischer-transformed correlation values.

Within the VS cortical network, two regions showed weaker functional connectivity with seed-to-ROI analysis in a-MCI compared with HC. The right middle frontal gyrus showed a significantly weaker connection to the left precentral gyrus, left middle frontal gyrus, and left superior frontal gyrus (Figure 3). Furthermore, the right superior frontal gyrus also demonstrated a weaker connection to the left precentral gyrus, left inferior temporal gyrus pars opercularis, and left temporal pole (Figure 4). We found stronger functional connectivity in a-MCI patients. The left inferior temporal gyrus showed stronger functional connectivity to the left middle frontal gyrus and left inferior frontal gyrus (pars triangularis) (Figure 5).

## Discussion

Our study involved 78 participants, 46 healthy controls and 32 who belonged to the multiple-domain a-MCI group. Our major findings are that in this patient group 1, VS skills are the most impaired cognitive subdomain; 2, thinning of the lateral temporal cortices is a characteristic hallmark; and 3, functional connectivity changes are present within the visuospatial network (reduction of commissural connectivity and increased connectivity between the left frontotemporal areas). The clear novelty of our report is that we showed characteristic changes in the VS network in MCI patients using resting-state fMRI indicating a potential new biomarker for the early recognition of cognitive decline.

Our neuropsychological results revealed that the a-MCI and healthy control groups significantly differed in MMSE scores with the HC group outperforming the a-MCI group. Our finding is in line with the current literature: studies agree that a-MCI patients show weaker performance on the MMSE test manifesting in lower scores (Petersen and Morris, 2005; Rami et al., 2007; Slavin et al., 2007). Studies also suggest that while a-MCI patients show weaker performance on the MMSE, the test does not reach the level of sensitivity required for accurate detection of the condition (Arevalo-Rodriguez et al., 2015). The two groups involved in our study significantly differed in ACE scores with the a-MCI group demonstrating weaker performance. Furthermore, the difference between the HC and a-MCI groups in ACE scores proved to be greater than in the MMSE scores (*F* = 11.065; *p* < 0.001 for ACE vs. *F* = 9.098; *p* < 0.001 for MMSE). This finding of ours is also in line with studies in this literature suggesting that ACE has a potential role in the detection of MCI with higher accuracy than MMSE (Fang et al., 2014; Csukly et al., 2016; Matias-Guiu et al., 2017). However, both MMSE and global ACE scores showed relatively low utility in the differentiation of amnestic and non-amnestic subtypes of MCI. Its importance lies in the fact that a-MCI shows a higher conversion rate to dementia than the non-amnestic subtype (Ahmed et al., 2008). By analyzing collectively the results of the various neuropsychological test used in this study, we could determine that our MCI patients belonged to the multiple-domain a-MCI subtype (Petersen and Morris, 2005) since they showed weaker performance in more than one cognitive domain including memory. The multiple-domain pattern of our cohort is reinforced by the analysis of TMT scores (where indicated cognitive abilities are relatively independent of memory functions) showing significantly weaker performance in a-MCI patients. This is in line with previous reports indicating the usefulness of TMT in the recognition of multiple-domain a-MCI (Terada et al., 2013; Csukly et al., 2016). Our analysis of cognitive domains represented by ACE subscores resulted in interesting findings. Despite that our patients belonged to the multiple-domain a-MCI category as described earlier, the only significant difference between the HC and a-MCI groups in ACE subscores (after correction for multiple comparisons) was detected in VS abilities (*F*-value is 8.32, corrected *p* < 0.001 vs. the *F* = 3.35, corrected *p* = 0.09 of memory). It confirms the validity of our hypothesis that the sensitive analysis of the VS network dysfunction might serve as a potential biomarker of a-MCI. When interpreting neuropsychological results, higher age in the MCI group must be considered since aging is associated with lower performance in cognitive tasks. However, ANCOVA analysis with age and sex set as covariates revealed that neither age nor sex had a significant modifier effect on the results of neuropsychology (*p* > 0.05) in our sample.

Plentiful studies are available on the assessment of VS skills in AD, and while fewer studies analyzed MCI studies with the same goal, they showed important results. Mapstone et al. (2003) demonstrated that one-third of MCI patients showed pervasive impairment of visual motion perception in correlation with poor performance on the Money Road-Map test. The study of O'Brien et al. (2001) concluded that VS impairment might represent the earliest sign of the neurodegenerative process and this idea was reinforced by critical reviews as well (Iachini et al., 2009). A recent study also suggested that VS assessment is a precise method for the detection of multiple-domain MCI (Wasserman et al., 2020). The exact diagnostic accuracy of VS skills was addressed in the study of Mitolo et al. (2013) showing strong discriminative power (AUC > 90) for the detection of MCI.

In this study, we applied structural and functional MRI to analyze cortical areas associated with VS function and the alteration of the VS network. Structural imaging results of our sample revealed a marginal reduction in the a-MCI group in cortical thickness in several cortical areas. However, after correction for multiple comparisons (corrected *p* < 0.05), only the differences in the left temporal pole and right superior temporal gyrus remained significant. Sex and age differences must be considered in the interpretation as well since aging is associated with cortical thinning. Regarding structural MRI results, higher age was associated with the decreased thickness of the left and right frontal areas and the left and right temporal areas (*p* < 0.05). However, the modifier effect of age disappeared after Benjamini–Hochberg correction (*p* > 0.05). Sex did not have a significant modifier effect in our sample (*p* > 0.05). Our results showing the discriminative role of the left temporal pole and right superior temporal gyrus thinning in the assessment of a-MCI are in line with this literature. A study with large samples such as the Alzheimer's Disease Neuroimaging Initiative (ADNI) showed that VS deficit indicates a-MCI adequately and the conversion of MCI to AD (Choi et al., 2020). In this experiment, structural MRI data were also analyzed and the results indicated that a-MCI patients were characterized by the predominant reduction of the temporal cortical thickness. It is supported by various studies showing that temporal areas including medial and lateral structures have the highest discriminating role in the detection of a-MCI (Karas et al., 2008; Xie et al., 2015; Csukly et al., 2016). In addition, some reports suggest the superiority of the lateral temporal lobe areas compared to medial temporal structures (Shen et al., 2011).

The remarkable changes in VS function are barely understandable solely from the sMRI findings. In our sample, significant reduction is detectable only in the thickness of the temporal pole and the superior temporal gyrus. While the temporal pole and superior temporal gyri are integrated parts of the VS network, many other areas have a mandatory role as well (e.g., calcarine cortex, posterior part of the superior parietal lobule, inferior temporal gyrus, occipitotemporal gyri, supramarginal gyrus, angular gyrus, caudal part of middle frontal gyrus, precentral gyrus, and insula) (Graaf et al., 2010). Interestingly, in the largest neuroimaging cohort (ADNI 2), VS impairment was associated predominantly with the thinning of the superior temporal gyrus (Choi et al., 2020). It reinforces the strength of our sample but also points to the importance of functional analysis.

In this study, all 32 multiple-domain a-MCI patients and 46 healthy control participants underwent resting-state fMRI acquisition. Our major findings reveal that there is increased functional connectivity in the a-MCI subject group within the left fronto-temporal network. At the same time, the right temporal areas have weaker connections to the left temporal and frontal areas. Contrary to Bonanni et al.'s findings (Bonanni et al., 2021), hyperconnectivity and decreased connectivity were not sequential findings in our study; the two phenomena were present simultaneously but affected different brain regions. It should be noted that we analyzed only a specific network, so conclusions cannot be drawn for the entire cortical activity. Changes in the activity in our sample might be the consequence of the altered VS network, where the widely distributed right hemispheric organizers of the VS functions lose their long-distance commissural connections and the left short-distance associative connections become more dominant to compensate for the functional damage. This is in line with diffusion tensor imaging studies showing the prominent loss of commissural connections in the earliest stages of MCI (Zhuang et al., 2010; Nir et al., 2013). With these resting-state fMRI findings, our study brings novelty to the literature. While numerous fMRI studies observed VS dysfunction in AD (Thulborn et al., 2000; Prvulovic et al., 2002; Bokde et al., 2010), only three studies analyzed the VS network in MCI (Vannini et al., 2007; Bokde et al., 2008; Alichniewicz et al., 2012). All previous fMRI studies conducted on MCI patients applied task-related data acquisition, while resting-state studies on the functional connectivity of VS network are absent. The three previously mentioned task-based fMRI studies reported increased activation in the left frontal areas in the MCI group. Since the right hemisphere seems to have a dominant role in VS processing (Ng et al., 2000; Corballis, 2003), all three studies concluded that the lower activity of the right hemisphere and increased involvement of the left during a VS paradigm is a compensatory mechanism of the MCI-related functional damage. Our conclusion is also in line with the conclusion of these three studies (Vannini et al., 2007; Bokde et al., 2008; Alichniewicz et al., 2012). Further studies are required to confirm our findings and to test their application in the recognition of the prodromal phase of cognitive decline in patients with subjective memory complaints or healthy individuals with high dementia risk.

Regarding the patient population, it is important to notice that this study was focused on multiple-domain a-MCI patients representing a heterogenous but high-risk patient population for dementia. These subtypes of MCI patients show the highest potential to convert into dementia, however, not into AD or into typical AD only (Petersen and Morris, 2005). Moreover, around 25% of AD cases present as atypical meaning that the localization of neurodegeneration and cognitive symptoms differ from typical AD. Atypical forms include the behavioral variant, logopenic primary progressive aphasia, the corticobasal variant, and the posterior cortical atrophy (Frisoni et al., 2022). One might argue that these atypical presentations of AD may influence the results of our study since the long-term outcome of these patients is not known at the moment. Posterior cortical atrophy as a possible outcome is especially intriguing due to the early but remarkable impairment of VS skills. It remains an open question and requires a follow-up on how prodromal decline in VS functions indicates the further risk for dementia.

## Conclusion

Amnestic-type MCI represents an important category of cognitive decline since these patients show the highest risk for conversion into dementia. MRI is a non-invasive and widely available technique for the precise detection of early cognitive impairment. In this study, we applied neuropsychology, s-MRI, and resting-state fMRI to compare a-MCI patients with control subjects. The strength of our study is the double-center arrangement, the rigorous statistical reporting (correction for multiple comparisons in all analyses and reporting of effect size), and the strict diagnostic regime of a-MCI. The limitation of the study is that patients are characterized by multiple-domain cognitive impairment; however, this category represents the highest risk for dementia within the MCI spectrum. A further limitation is that patients and controls showed significant differences in age and sex distribution. While corrections were applied and individual effects of sex and age have been analyzed, differences might modify the interpretation of our results. To our knowledge, our study is the first to analyze the functional connectivity within the VS network using resting-state data in a-MCI.

In our multiple-domain a-MCI patient cohort, VS impairment was the most dominant feature of cognitive decline. With a strict statistical correction, we also showed that reduction of the thickness of the temporal pole and the superior temporal gyrus is the most characteristic structural hallmark of a-MCI. The analysis of the VS network with fMRI demonstrated increased functional connectivity between the left fronto-temporal areas, while connectivity was reduced between the right and left frontal structures. It might correspond to the compensatory remodeling of the VS network. Since the resting-state setting simplifies the possible automatization of data analysis, detection of VS system alterations might provide a non-invasive, fast, and sensitive biomarker for the early recognition of cognitive decline.

## Data Availability Statement

The raw data supporting the conclusions of this article will be made available by the authors, without undue reservation.

## Ethics Statement

The studies involving human participants were reviewed and approved by Hungarian Medical Research Council. The patients/participants provided their written informed consent to participate in this study.

## Author Contributions

DB was responsible for the neuropsychological assessment of patients and statistical analysis. JZ controlled the patient recruitment on behalf of NIMNN. TW performed the literature research and defined the MRI network. MK analyzed the MRI-s. AD was responsible for neuroimaging data acquisition. AK and AH were responsible for study design and the concept of the study. GC controlled the experiments on behalf SU. All authors contributed to the article and approved the submitted version.

## Funding

This study was supported by the National Brain Research Program I, II (KTIA_NAP_13-1-2013-0001 and 2017-1.2.1-NKP-2017-00002), the Hungarian Scientific Research Fund 2019 of the National Research, Development and Innovation Office (PD- 132652), the Janos Bolyai Research Scholarship of the Hungarian Academy of Sciences (bo_78_20_2020), and the Development of scientific workshops in medical, health sciences and pharmacy training (EFOP-3.6.3-VEKOP-16-2017-00009). This is an EU Joint Programme–Neurodegenerative Disease Research (JPND) project. The project was supported through the following funding organization under the aegis of JPND-www.jpnd.eu (National Research, Development and Innovation, Hungary, 2019-2.1.7-ERA-NET-2020-00006).

## Conflict of Interest

MK was employed by Siemens Healthcare. The remaining authors declare that the research was conducted in the absence of any commercial or financial relationships that could be construed as a potential conflict of interest.

## Publisher's Note

All claims expressed in this article are solely those of the authors and do not necessarily represent those of their affiliated organizations, or those of the publisher, the editors and the reviewers. Any product that may be evaluated in this article, or claim that may be made by its manufacturer, is not guaranteed or endorsed by the publisher.
